# Acupuncture for chronic pelvic inflammatory disease: A systematic review protocol

**DOI:** 10.1097/MD.0000000000010225

**Published:** 2018-03-30

**Authors:** Ying Cheng, Youcai Yuan, Yuhao Jin, Na Xu, Taipin Guo

**Affiliations:** a Yunnan University of Traditional Chinese Medicine, Kunming, Yunnan Province; b Shaanxi University of Traditional Chinese Medicine, Xianyang, Shaanxi, China.

**Keywords:** acupuncture, chronic pelvic inflammation disease, chronic pelvic pain, depression, protocol, systematic review

## Abstract

**Background:**

Chronic pelvic inflammation disease (PID) is a difficult-to-treat gynecological disorder with complex etiologies. Acupuncture has been applied widely for treating chronic pelvic inflammation or chronic pelvic pain symptoms in China. The aim of this review is to undertake a systematic review to estimate the effectiveness and safety of acupuncture on chronic PID.

**Methods:**

A literature search will be conducted electronically with date up to October 2018 in MEDLINE, Cochrane Library, EBASE, and CNKI databases, using combination subject terms of chronic pelvic pain (or chronic pelvic inflammation, and chronic pelvic pain symptoms, etc.) and acupuncture related treatment. Also duplicates will be removed. The primary outcomes consisted of improvement rate and pain relief. Secondary outcomes include the recurrence rate and side effects, such as pneumothorax, bleeding, serious discomfort, subcutaneous nodules, and infection. Systematic reviews and databases will be searched for randomized controlled trials on acupuncture for chronic PID with acupuncture treatment will be included. Cochrane RevMan V5.3.5 risk of bias assessment tool will be implemented for risk of bias evaluation, data synthesis, meta-analyses, and subgroup analysis while condition is met. Mean difference (MD), standard mean difference (SMD), and dichotomous data will be used to present continuous outcomes.

**Results:**

This study will generate a comprehensive review of current evidence of acupuncture for chronic pelvic inflammation diseases.

**Conclusion:**

The study will provide updated evidence to evaluate the effectiveness and side effects of acupuncture for chronic pelvic inflammation disease.

**PROSPERO registration number:**

CRD42018087950.

## 1 Introduction

### 1.1 Description of the condition

Chronic pelvic inflammatory disease (PID)^[1–3]^ is common dysfunction characterized by lower urinary tract symptoms and abdominal pain. It is easy to relapse after treatment, and up to one-third of women with PID^[4]^ could develop chronic pelvic pain. In China, chronic PID is also a common disease in traditional Chinese medicine (TCM) gynecological clinic, accounting for 20% of all outpatient appointments in women's health services.^[5]^ Moreover, estimated 4% to 20% of women between the ages of 15 and 50 experience chronic pelvic inflammatory disease (CPID).^[6]^ In the United States, PID is estimated to occur in over 750,000 US women annually and generates annual costs of around US$4.2 billion^[7]^ for healthcare.

About 25% of women with PID will experience long-term sequelae,^[8]^ including annexitis, irregular menstrual, infertility, ectopic pregnancy, or chronic pelvic pain. Further, women suffering from PID have a 6 to 8 risk of infertility.^[9]^

According to the US Centers for Disease Control and Prevention (CDC) guidelines,^[10,11]^ PID is a clinical condition involves infection of microorganisms from reproductive system to contiguous tissues. It also comprises complicated inflammation of annexitis^[12]^, salpingitis^[13]^, and pelvic peritonitis.^[14]^ Therefore, diagnosis of PID requires a careful analysis of the history and a thorough medical examination. Most cases of PID result from incomplete and delayed treatments of acute pelvic inflammation. The etiological factors may include polymicrobial infection, pelvic musculoskeletal disorders, and psychoneurological causes.^[15]^ According to a GP's guide to PID,^[16]^ the effective diagnostic method is laparoscopy, which shows inflamed fallopian tubes in PID. In clinic, PID should be suspected when low abdominal pain is associated with adnexal or uterine pain. In this case, pelvic ultrasonography is necessary to exclude ovarian abscess.^[17]^ Microbiological diagnosis involves vaginal and endocervical sampling for bacteriological analysis. And the presence of many poly-microbe supports a diagnosis of reproductive system infection.^[18]^ In general, sexually acquired chlamydia trachomatis and Neisseria gonorrhoeae^[19]^ are common causes. The infection and inflammation of the reproductive organs (the uterus, fallopian tubes, and ovaries) can then transmit to lower genital tract structure.^[20]^ Untreated PID^[21]^ can lead to serious consequences including irregular menstruation, tubal occlusion infertility, pelvic adhesion syndrome, pelvic congestion syndrome, abscess formation, etc.

Meanwhile, many cases of PID are reported as functional or psychosomatic disorders.^[22]^ And the cause has never been found. Although presentations vary according to the cause and severity of the disease, it is commonly characterized by noncyclical abdominal pain, dysmenorrhea, lumbosacral pain, and lingering leucorrhea, etc.^[23]^

Although the etiology and pathogenesis are still unclear, many clinical practice guidelines have been developed worldwide. The International Union against Sexually Transmitted Infections (IUSTI) and the CDC has recommended antibiotic therapies,^[24]^ which cover Chlamydia trachomatis, Neisseria gonorrhoeae, and anaerobic bacteria, administered intravenously, intramuscularly, or orally. However, PID is polymicrobial in etiology.^[25]^ So a disadvantage of this strategy is technical problems that limit the possibility of specific bacteria culture from deep pelvic infections. Although broad-spectrum combination antimicrobial therapy is recommended, uncertainties regarding the effectiveness of antimicrobial therapy exist. Further, there has been accumulating evidence that antibiotics potentially have a number of undesired side effects including gastrointestinal disorders, cutaneous reactions, the potential severity of hepatic side effects and it can produce resistant variants^[26]^; for surgery, patients with PID are not routinely referred for physical therapy until the condition is found to be resistant to antibiotic therapy.^[27]^ Meanwhile, surgical operations have been reported of little benefit, and indications for surgery should be carefully considered to avoid iatrogenic damage in uterus; for other routine therapies of tranquilizers and hormone, they can only relieve symptoms for short term and lack of long-term follow-up.^[28]^

Obviously, although therapies of antibiotics, surgery, and hormone have been tested to be effective in short term, full evaluation of recurrent rate and the risks of long-term squeal is not available. In general, current medical treatment cannot give permanent therapeutic effects for chronic PID.^[29]^ As a result, the patient may suffer from repeated attacks, making the disease intractable.

### 1.2 Description of the intervention

Considering the side reaction in antibiotics and the high risk and narrow use of surgery, more clinicians have applied complementary and alternative therapy including TCM to cure chronic pelvic inflammation disease.^[30,31]^

Acupuncture has been widely used in Chinese clinical practice. It uses very fine needles to stimulate specified acupuncture points. If qualified acupuncturists perform it properly, acupuncture is a safe treatment without any side effects. Acupuncture is used to treat a range of conditions, such as myofacial pain, muscle disorders, and neurological disorders.^[32,33]^

A study showed there were 1088 articles of chronic PID using TCM in CNKI database by 2012, and most of the methods were herbs and acupuncture.^[34–37]^

### 1.3 How the intervention might work?

Acupuncture provides overall coordination, helping to achieve the state of relative equilibrium of body and mind. Guided by the TCM theory of differential diagnosis and treatment,^[38,39]^ we believe chronic pelvic inflammation is mostly caused by invasion of pathogenic heat or dampness and blood circulation disorder leading to lower abdominal pain and other symptoms. Acupuncture is reported to invigorate blood circulation, relieve pain, prevent and treat hemorheologic disorder.^[40,41]^

### 1.4 Why it is important to conduct this review?

Characterized by easy operation, durable and strong stimulation, and long interval between each treatment, acupuncture has been broadly used to treat chronic pelvic inflammation.^[42–44]^ It is an easy applicable method without side effect in long-term sequel. The needle can work as an effective painkiller, invigorate the channel-*qi*, remove the tissues, and generate the new.^[45,46]^ Despite lack of effectiveness evaluated and normative management plan, most Chinese TCM hospitals have conducted acupuncture to treat chronic PID based on their own experience. To estimate the safety and effect appeared to be especially important, and it is also necessary to provide a treatment suggestion based on current evidences.

### 1.5 Objectives

The primary aims of this systematic review are to estimate the effectiveness and safety of acupuncture on chronic PID and formulate a treatment suggestion. We will focus on treating the pain itself and the underlying cause. Combination of drug therapy with medications with different mechanisms of action may improve therapeutic results.

## 2 Methods

### 2.1 Study registration

PROSPERO registration number is CRD42018087950. This protocol report is structured according to the Preferred Reporting Items for Systematic Reviews and Meta-Analyses Protocols (PRISMA-P) statement guidelines.^[47]^

### 2.2 Inclusion criteria for study selection

#### 2.2.1 Types of study

To evaluate the curative effects of acupuncture on chronic PID, this review is confined to RCTs comparing acupuncture with a control group, which contained drug, no treatment, placebo, diet and exercise therapy, and other types of acupuncture including fine needles, fire needling, electronic needling, ear auricular pressure treatment, acupoint pressure, and so forth. It is deemed a randomized study if the trial stated the “randomization” phrase, and the blinding is not restricted. Besides, Chinese and English publications are the limitation of language. The animal mechanism studies, case reports, self-pre- and postcontrol, or non-RCTs are excluded.

#### 2.2.2 Types of participants

It included the participants with no limitation of age, type of chronic PID, including specified and unknown causes. The definitions of chronic pelvic inflammation disease or chronic pelvic pain, or chronic abdominal disorder are included. Patients with acute medical conditions or pregnancy are excluded.

#### 2.2.3 Types of intervention

The review comprises clinical trials with the treatment of acupuncture. We will study the types of acupuncture including fine needle, floating needle, electro-acupuncture, let bleeding, acupoint injection, fire needle, needle knife, and acupressure. Studies to compare the effect of different acupuncture therapies will be excluded.

#### 2.2.4 Types of outcome measures

The primary outcomes consisted of improvement rate and pain relief. Secondary outcomes include the recurrence rate and side effects, such as pneumothorax, bleeding, serious discomfort, subcutaneous nodules, and infection. Systematic review and will be performed independently.

### 2.3 Data sources

A literature search will be conducted up to October 2018 in the databases of MEDLINE, Cochrane Library, EBSCO, Web of Science, EBASE, Springer, WHO International Clinical Trials Registry Platform (ICTRP), CNKI, Wanfang, CBM, and VIP. The item of RCT is also chosen in the corresponding databases in the languages of Chinese and English.

### 2.4 Search strategy

The following search keyword or combination subject terms are used: RCT (controlled clinical trial); acupuncture (e.g., “acupuncture” or “body acupuncture” or “scalp acupuncture” or “manual acupuncture” or “auricular acupuncture”, and “electro- acupuncture” or “fire needling”; chronic pelvic inflammation disease; chronic pelvic pain (e.g., “chronic pelvic pain” or “chronic pelvic ache” or “chronic pelvic disorder” and “chronic pelvic illness”); and chronic abdominal disorder. For Chinese databases, these searching terms will be accurately translated. The search strategies for Medline are summarized in Table 1.

**Table 1:**
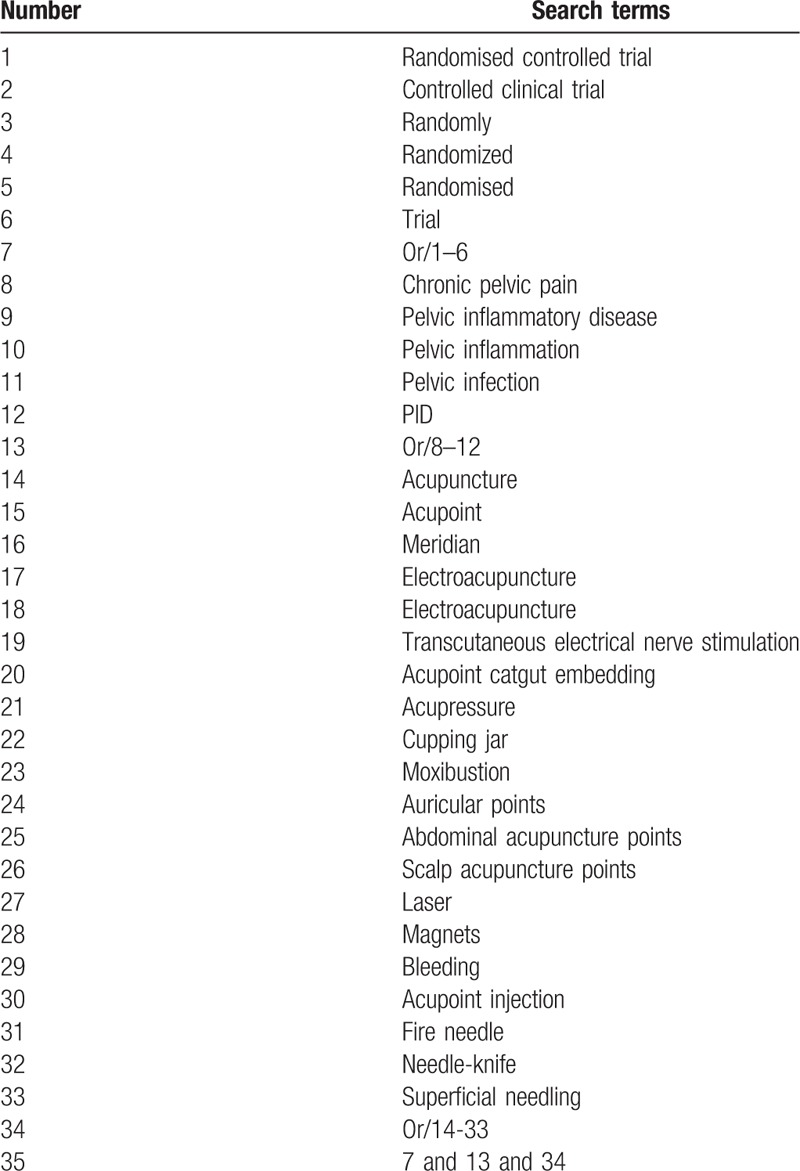
Medline search strategy.

### 2.5 Data collection and analysis

#### 2.5.1 Selection of studies

**Figure 1:**
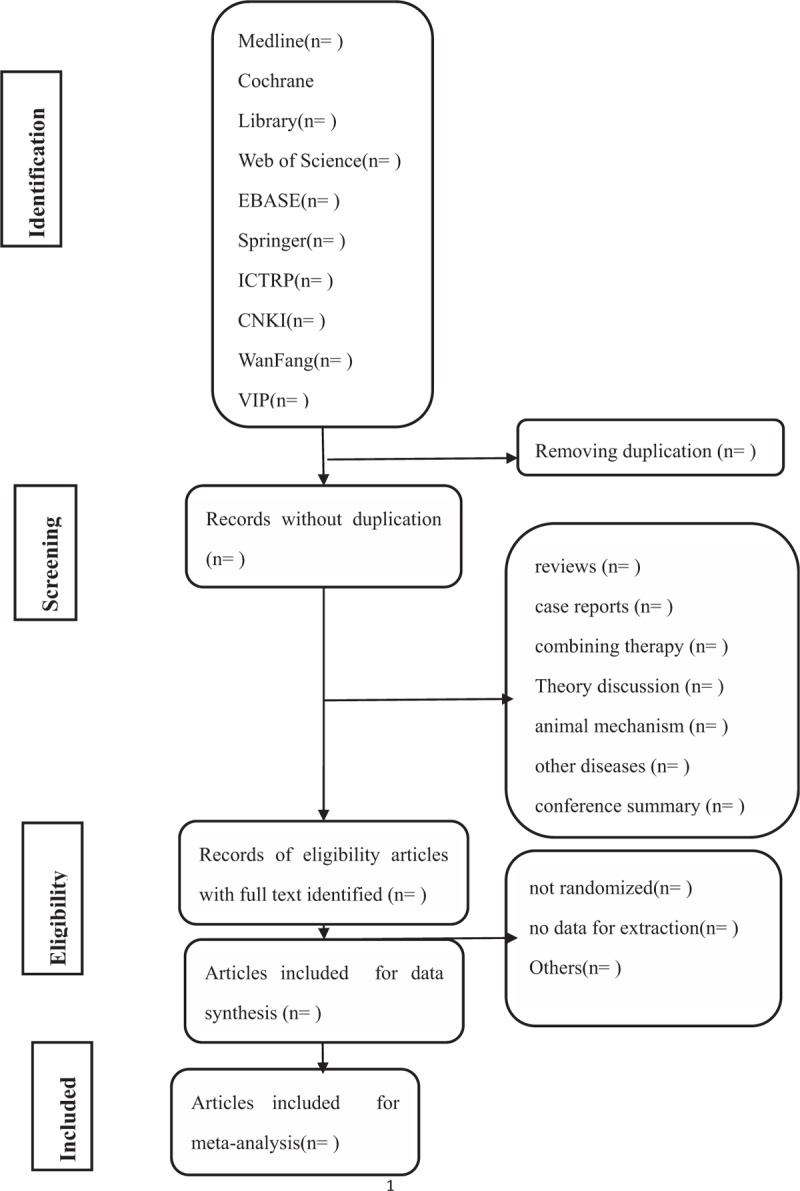
Flow diagram of studies identified.

Each literature of title and abstract is scanned by 2 reviewers (YC and YCY) who have been trained and gained certifications in Chinese Cochrane Centre. All relevant articles of full text are investigated. When the 2 reviewers cannot agree on the selection process through consultations, the third reviewer (TPG) will ultimately make the decision. The primary selection process is shown in a PRISMA flow chart (Fig. 1)

#### 2.5.2 Data extraction and management

The extracted information includes descriptions of studies, characteristics of participants, interventions of both the observation group and control group, quality, randomization, allocation concealment and blinding methods, outcome measures, main outcomes, adverse effects, duration of follow-up, type and source of financial support, and the Standards for Reporting Interventions in Controlled Trials of Acupuncture (STRICTA) checklist.

#### 2.5.3 Assessment of risk of bias and reporting of study quality

Risk of bias is used to evaluate the quality of study with the Cochrane Collaboration's risk-of-bias assessment method and complete the STRICTA checklist for the included studies. The decision of risk is made by 2 reviewers (YHJ and NX). If inconsistent results appear, the final decisions will be made by the third author (TPG). For missing or ambiguous data, we will try to contact the author as possible, and for duplicate publication we only select the original.

#### 2.5.4 Measures of treatment effect

Mean differences (MDs) with 95% confidence intervals (95% CIs) will be used to analyze continuous data. Other forms of data will be changed into MD values. Risk ratio with 95% CIs will be used to analyze dichotomous data. If significant heterogeneity is detected, a random-effects model will be used.

#### 2.5.5 Unit of analysis issues

The analysis will focus on patients in randomized studies. If more than one acupuncture objective is used, we will conduct separate multiple meta-analyses for each treatment objective. If multiple nonacupuncture control groups are included, pooled analyses of the control groups against the intervention group will be used.

#### 2.5.6 Management of missing data

If there are missing or incomplete data for the primary results, we will contact the corresponding authors for the missing data. If the missing data cannot be obtained, it will be excluded from analysis.

#### 2.5.7 Assessment of heterogeneity

Review Manager (version 5.1, the Nordic Cochrane Centre, Copenhagen, Denmark) is applied to assess curative effect and publication bias. Forest plot is used to illustrate the relative strength of curative effect. Meanwhile, the funnel plot will picture the publication bias visually as the number of trials is more than 10. If significant heterogeneity is detected, a random-effects model will be used.

#### 2.5.8 Assessment of reporting biases

If more than 10 trials are included, funnel plots will be used to assess reporting biases. If funnel plot asymmetry is detected, the reasons will be analyzed.

#### 2.5.9 Data synthesis

We will use RevMan for all statistical analyses. If considerable heterogeneity is observed, a random-effects model with 95% CIs will be used to analyze pooled effect estimates. If necessary, subgroup analysis will be performed with careful consideration of each subgroup.

#### 2.5.10 Subgroup analysis

A subgroup analysis will be performed according to control intervention and different outcomes.

#### 2.5.11 Sensitivity analysis

A sensitivity analysis will be performed according to the following criteria: sample size, heterogeneity qualities, and statistical model (random-effects or fixed-effects model).

## 3 Discussion

Chronic PID comprises symptoms of pain and disorders in the lower abdomen (uterus, ovaries, fallopian tubes, and cervix). Sequelae may include fever, irregular menstruation, sterility, immunological dysfunction, and mental disorders.^[48]^

Potential etiologies for chronic PID may vary^[49]^ individually. Proved causes include polymicrobial infection, nonbacterial infection, autoimmunity, and pelvic floor muscle dysfunction. And many cases are diagnosed with unknown pathogen.

Having the effect of promoting metabolism circulation, balancing, improving immunity, purifying meridian, and harmonizing *qi* and blood,^[50,51]^ traditional Chinese acupuncture has shown to be effective and safe with potent operability, low cost, and, safety, especially suitable for chronic PID results from nonbacterial infection, autoimmunity, neurological dysfunction and pelvic floor muscle dysfunction and with unknown causes. Many chronic PID cases are reported with the clinical diagnosis of blood circulation problem in different degree. Acupuncture has been proved to stimulate blood circulation, improve permeability of the cellular membranes, and accelerate absorption of inflammation.^[50]^ Therefore, it can be used to treat chronic and refractory pelvic disorders.^[51]^

A present review suggests that a significantly positive benefit is obtained from the administration of acupuncture therapy.^[52]^ The use of acupuncture for symptoms of chronic PID symptoms should be considered, although further evidence from placebo controlled RCTs is required.

According to Cochrane method, this research is based on the existing clinical RCT evidence analysis at home and abroad, retrieving and screening the main electronic literature database with evidence of evidence-based medicine, to better guide clinical practice.

## Author contributions

**Conceptualization:** T. Guo, N. Xu, T. Guo, Y. Chen.

**Methodology:** N. Xu, Y. Yuan.

**Software:** T. Guo.

**Supervision:** T. Guo.

**Validation:** T. Guo, Y. Jin.

**Writing – original draft:** T. Guo, Y. Chen, Y. Yuan.
